# Peer-to-Peer Contact Tracing: Development of a Privacy-Preserving Smartphone App

**DOI:** 10.2196/18936

**Published:** 2020-04-07

**Authors:** Tyler M Yasaka, Brandon M Lehrich, Ronald Sahyouni

**Affiliations:** 1 Department of Otolaryngology – Head and Neck Surgery University of California, Irvine Irvine, CA United States; 2 Department of Biomedical Engineering University of California, Irvine Irvine, CA United States; 3 Medical Scientist Training Program University of California, Irvine Irvine, CA United States

**Keywords:** COVID-19, smartphone, privacy, contact tracing, peer-to-peer, epidemic, personal data, mobile phone, coronavirus, pandemic

## Abstract

**Background:**

The novel coronavirus disease 2019 (COVID-19) pandemic is an urgent public health crisis, with epidemiologic models predicting severe consequences, including high death rates, if the virus is permitted to run its course without any intervention or response. Contact tracing using smartphone technology is a powerful tool that may be employed to limit disease transmission during an epidemic or pandemic; yet, contact tracing apps present significant privacy concerns regarding the collection of personal data such as location.

**Objective:**

The aim of this study is to develop an effective contact tracing smartphone app that respects user privacy by not collecting location information or other personal data.

**Methods:**

We propose the use of an anonymized graph of interpersonal interactions to conduct a novel form of contact tracing and have developed a proof-of-concept smartphone app that implements this approach. Additionally, we developed a computer simulation model that demonstrates the impact of our proposal on epidemic or pandemic outbreak trajectories across multiple rates of adoption.

**Results:**

Our proof-of-concept smartphone app allows users to create “checkpoints” for contact tracing, check their risk level based on their past interactions, and anonymously self-report a positive status to their peer network. Our simulation results suggest that higher adoption rates of such an app may result in a better controlled epidemic or pandemic outbreak.

**Conclusions:**

Our proposed smartphone-based contact tracing method presents a novel solution that preserves privacy while demonstrating the potential to suppress an epidemic or pandemic outbreak. This app could potentially be applied to the current COVID-19 pandemic as well as other epidemics or pandemics in the future to achieve a middle ground between drastic isolation measures and unmitigated disease spread.

## Introduction

The novel coronavirus disease 2019 (COVID-19) global pandemic represents an urgent public health crisis. According to epidemiologic modeling conducted by Ferguson et al [1], both the United States and the United Kingdom face a dilemma in terms of choosing a public health response. On one hand, the models predict severe consequences, including high death rates, if the virus is permitted to run its course without any intervention or response. However, the authors conclude that an optimal outcome following a strategy of disease suppression would likely require dramatic alterations to daily life, including social distancing for the entire population until a vaccine is available. Such an intervention may result in significant economic loss. Ferguson and colleagues [1] noted that technological solutions such as a contact tracing smartphone app may provide alternatives to the drastic measures they proposed.

Contact tracing is the process of tracing potential transmission routes of an infection through a population for the purposes of isolating those who may have been exposed and reducing further transmission. Contact tracing in varying forms has been used for several diseases including tuberculosis, HIV, and Ebola infection [2,3]. Smartphone-based contact tracing presents a viable solution to limiting disease transmission; however, such an app presents significant concerns regarding privacy.

Recent events such as the Equifax security breach and the Cambridge Analytica controversy regarding data collection through Facebook have highlighted the privacy concerns regarding the use of personal data. These concerns are especially pertinent in a health care setting [4-6]. Existing contact tracing apps typically rely on the collection of personal data such as timestamped locations to determine exposure risk [3]. Location data are highly personal, and the privacy concerns detailed above are especially salient for location data [7]. Furthermore, location is only a proxy for contact, and inferences about exposure based on location may not always be accurate due to noise in the data [8].

The balance between privacy and other objectives is certainly controversial, but there is an additional concern in the case of a contact tracing app. As we will demonstrate in this study, the efficacy of a contact tracing app depends on its adoption rate. Even those that may not prioritize privacy should be concerned about the efficacy of a contact tracing app that is believed by many to be an invasion of privacy. If a sufficiently large portion of a population does not participate due to privacy concerns, such an intervention may have limited impact on the outcome of a pandemic.

We propose a novel method for contact tracing using a smartphone app without the use of location data. The objective of this app is to provide an effective contact tracing mechanism without compromising user privacy.

## Methods

### Novel Proposal for Tracing Possible Routes of Transmission

At the core of our approach is a data structure, which we will call the *transmission graph*. The transmission graph consists of nodes, which represent *contact points* between individuals, and directed edges, which represent *transmission vectors* between contact points. Whenever an individual participates in a contact point, a transmission vector is added to the transmission graph from the individual’s prior contact point to the current contact point. The transmission graph, then, is a network of interactions between individuals. In this graph, each node (contact point) represents a physical interaction between two or more individuals at a specific time and place, during which microbial agents could potentially be transmitted from one individual to others. Of note, there are no entities in the graph that represent an individual, and location information is never encoded in this data structure. These properties of the transmission graph are fundamental to its privacy-preserving nature.

Each contact point (node) in the transmission graph can be in one of two states: *status positive* or *status unknown*. A status positive contact point is one that has been flagged as having one or more participating individuals that were positive for infection at the time of interaction. A status unknown contact point is simply one that has not been marked status positive; it is unknown whether any participating individuals were positive for disease at the time of the interaction. Status positive and status unknown are merely internal states of the graph. As we will demonstrate, they are distinct from the actual states that will be displayed to the end users.

**Figure 1:**
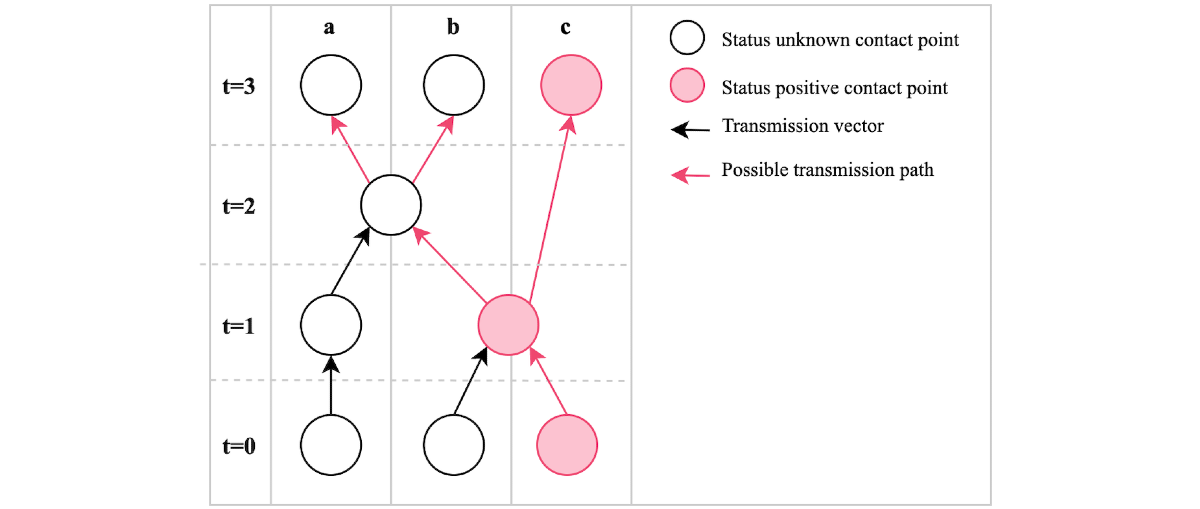
A network of interactions over time represented as a transmission graph. The rows represent units of time, and the columns represent individuals. By time t=3, all individuals have contact points with possible transmission paths. t: time point.

Using the simple data structure of the transmission graph, *possible transmission paths* can be determined for any given target contact point. A possible transmission path is defined as a path from a status positive node to a given target node. In other words, a transmission path is a sequence of transmission vectors that could be carrying microbial agents from a reported point of exposure. For any given target node, there may be 0, 1, or multiple possible transmission paths. An illustration of a simple transmission graph is provided in Figure 1.

### Smartphone App

We have developed a proof-of-concept smartphone app using the transmission graph data structure to implement peer-to-peer contact tracing. The app code is open source and publicly available on GitHub [9].

In the current prototype, users are assigned one of two risk levels: standard or elevated. The user is considered to have an elevated risk level if they have any recent contact points with possible transmission paths, and a standard risk level otherwise. “Recent” refers to some predetermined time, during which individuals who may have been exposed should take extra precautions, which we will call the *safety period*. Based on the protocol recommended by the World Health Organization (WHO) for quarantining individuals who have been in contact with a patient with confirmed COVID-19, we have set this time to 14 days in the current version of the app [10].

Possible transmission paths can also be assigned a *maximum length*, limiting the extent that a single reported diagnosis can raise the risk level of other users. A maximum length of 1, for example, would mean that only individuals that directly interacted with an infected individual would be assigned elevated risk levels. In the current app, the maximum length for transmission paths is set to 3.

When a user receives and reports a diagnosis, it is assumed that the infection was present for some time prior to the diagnosis. The app will determine the earliest contact point that occurred before this time and report it to the server accordingly. The amount of time that a user is assumed to have been infected is another parameter (*diagnosis delay*) that can be adjusted according to data and expert opinion. It is currently set to 2 days in accordance with the abovementioned WHO protocol.

### Simulation Model

Rigorous mathematical models of contact tracing have been previously described in the literature, and we did not attempt to replicate them here [11,12]. Rather, we developed a low-fidelity computer simulation model that facilitates disease spread through interaction of individuals at contact points across time, allowing for the explicit modeling of the transmission graph structure we have proposed here. With this model of disease spread, we can compare outbreak trajectories both with and without peer-to-peer contact tracing as we have described. Such a model, while not intended to describe real-world trajectories, allows for the demonstration of the feasibility of our proposal and provides a rudimentary mechanism to compare various scenarios and app parameters, such as the adoption rate, the diagnosis delay estimated by the app, and the safety period used by the app. The model was written in the R (R Foundation for Statistical Computing) programming language, and the source code is publicly accessible on GitHub [9]. Additionally, a public, web-based interface for the model is provided [13].

The model is based on the susceptible, infected, and recovered epidemiological model, [14,15] where each individual is considered to be in one of the *susceptible*, *infected*, or *recovered* states. However, unlike many models, the nodes in our graph are *contact points*, not individuals. This format explicitly captures the spread of disease across time, and contact points can be arranged in layers to visualize spread across time, analogous to the illustration in Figure 1. Individuals may move to a new contact point at each unit of time, forming a directed edge from the prior to the current contact point. Individuals may also refrain from being at a contact point at any point in time, to model home isolation.

The time between infection and awareness of infection (*diagnosis delay*) is a parameter of the model. Similarly, individuals are in the *infected* state for a time period according to the *infectious period* model parameter. Individuals may transmit disease to each other at the same contact location in a point in time according to the *transmission rate* parameter.

When contact tracing is enabled, a separate graph is generated, containing only the information that would be available to individuals using the peer-to-peer contact tracing app. This graph is the transmission graph we have described. At the beginning of the simulation, each individual is designated as either adopting the app (*participating*) or not adopting the app (*abstaining*), according to some probability specified by a model parameter (the *adoption rate*). Individuals that have adopted the app will perform three additional actions, in addition to the standard model behavior: (1) Participating individuals will log their diagnosis of infection onto the transmission graph, at their earliest contact point within the *estimated diagnosis delay* (a model parameter); (2) these individuals will log all their contact points and movements between contact points onto the graph; and (3) participating individuals will self-isolate if a path between a status positive contact point and a recent contact point exists, where “recent” is defined as those contact points within the time frame specified by the *safety period* parameter.

Thus, our model allows for the comparison of simulations both with and without use of a contact tracing app, as well as between different adoption rates in the population.

## Results

### Smartphone App

**Figure 2:**
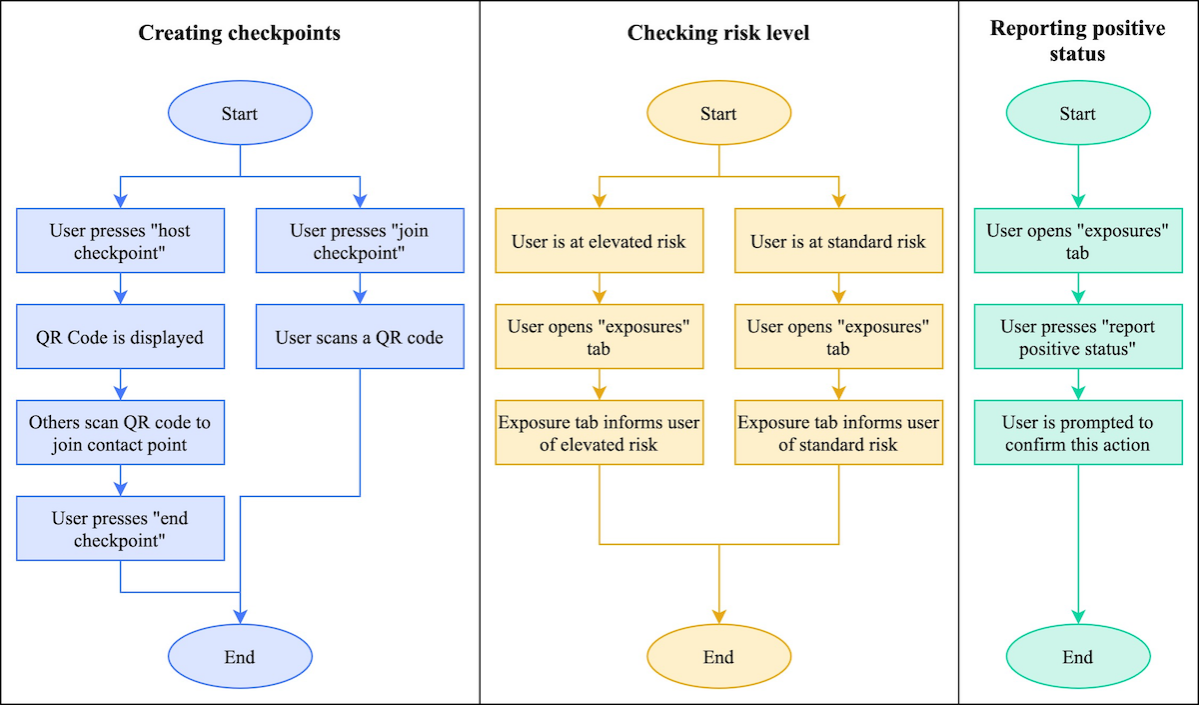
User flow diagrams for the three primary user flows in the peer-to-peer contact tracing app: creating checkpoints, checking risk, and reporting positive status. QR: Quick Response.

In the current app prototype, there are three primary user flows: creating contact points (termed “checkpoints” in the user interface), checking risk level, and reporting positive status. A diagram of these user flows is provided in Figure 2.

To create a checkpoint, a user may either host a new checkpoint or join an existing one. The host of a checkpoint is given a QR (Quick Response) code that is displayed on this user’s screen; other users may join the checkpoint by scanning the QR code. Creating and joining checkpoints are intended to be frequent activities for users of the app; a checkpoint should ideally be created for any gathering or interaction where disease transmission could occur, and all who participate in the gathering or interaction should join the checkpoint. Thus, checkpoints would not only be created for interactions among friends, but also for public gatherings at places such as restaurants and grocery stores. At public gathering places, a device with the checkpoint QR code could be made available for users to scan at the entrance.

In the current app prototype, users may check their risk level by opening the app. The “exposures” tab displays the user’s risk level, denoting whether any possible routes of transmission have been identified. Additionally, when the user has an elevated risk level, a banner is displayed at the top of the screen in the other tabs to make this information prominent. The app currently implements polling on a 30-second interval to retrieve the updated risk level from the server while the app is open. Future versions of the app may implement push notifications so that users are alerted when their risk level becomes elevated.

To report a diagnosis, users may press a button in the “exposures” tab. They are prompted to confirm this action before the server is notified. The app does not store any state changes associated with this action; this sacrifices good usability design, but it enhances the security and privacy of the user’s health data by not retaining any record of the self-reported diagnosis.

### Simulation Model

**Figure 3:**
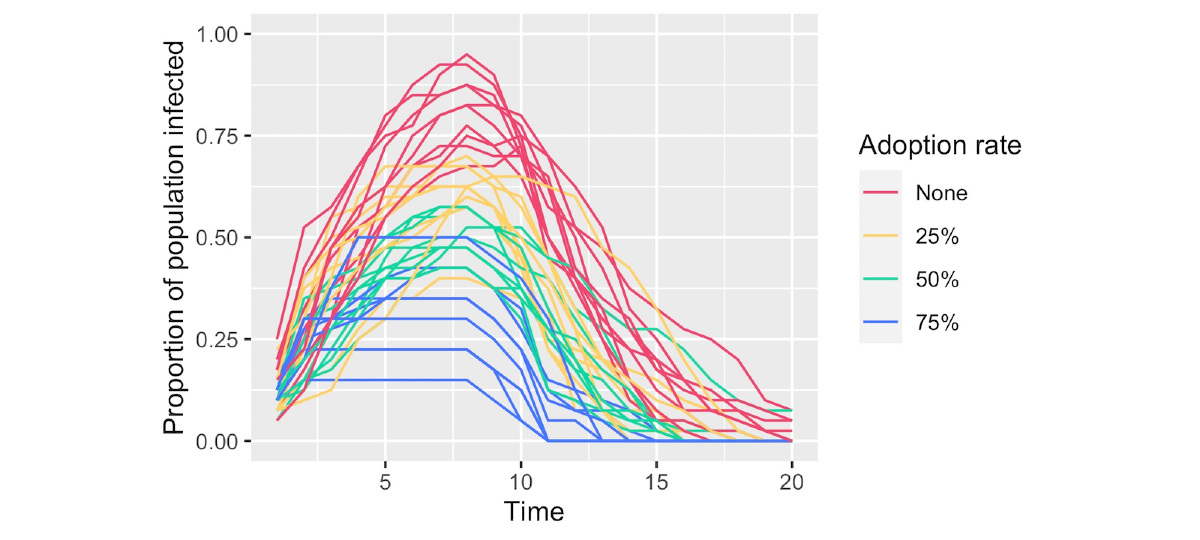
Comparison of infection curves from simulations at varying rates of peer-to-peer contact tracing application adoption. The proportion of the population with active infection is plotted across time for multiple adoption rates. Time is an arbitrary unit that represents the sequence of events in the simulation. The results of 10 random simulations per adoption rate are given.

A comparison of simulations produced by our simulation model with varying levels of adoption is presented in Figure 3. Our results demonstrate that adoption rate is key to the impact that such an app could have on the extent of an outbreak. Visually, even a 25% adoption would provide some suppression of the infection curve compared to no adoption. However, more substantial improvements on the trajectory of the outbreak are observed at higher levels of adoption.

## Discussion

### Information Access

**Figure 4:**
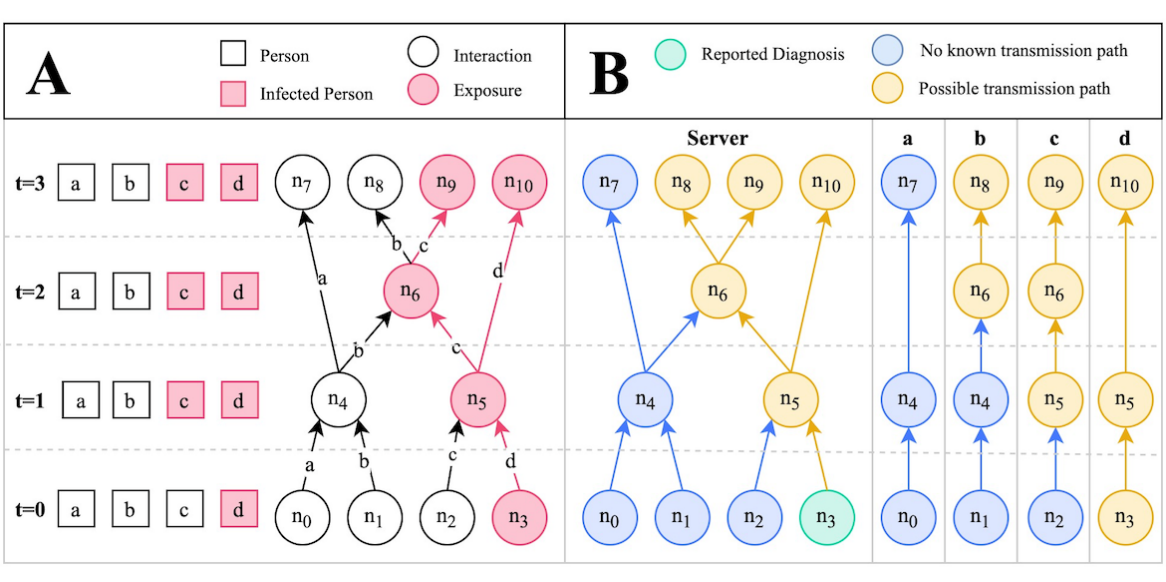
Disease spread scenario modeled as a transmission graph. (A) Graphical representation of a disease spread scenario across time. Contact points with infected individuals are denoted as exposures. Uninfected individuals may become infected at exposure points according to some probability (the transmission rate); hence, b does not become infected at n6. (B) Transmission graph corresponding to the scenario in A, depicting the information that is available to the server and each individual’s smartphone app. Only one node, n3, is associated with a reported diagnosis. The infection risk level at the other contact points can be inferred by checking for possible transmission paths. n: node; t: time point.

For the purposes of the smartphone app we are proposing, a key concept is the storage of and access to information. In the current prototype, all transmission graph data as described above is stored on a centrally managed server. Importantly, no user registration is required, and no personal information is collected; thus, the data on the server is anonymized. Users of the smartphone app will only be able to access their own checkpoints and whether there are any possible transmission paths to those checkpoints. An illustration of a hypothetical disease spread scenario, along with the corresponding transmission graph and the information available to the server and each user, is provided in Figure 4.

### Peer-to-Peer Design

Another important characteristic of the proposed app is its peer-to-peer nature. Traditional contact tracing apps often rely on a central entity to monitor individuals, their infection status, and their locations [3,16]. Our proposal does not require or use such entities. Instead, individuals rely on the joint participation of their peers, both in terms of creating contact points and reporting diagnoses. For the purposes of restricting manipulation of the system through false reports, we have introduced a centralized system for administering confirmation codes to users with confirmed diagnoses; this system will be described shortly. A centralized system for this purpose was introduced because such a system provides the only mechanism by which it is possible to confirm diagnoses. Nonetheless, only the generation of confirmation codes is centralized. The network of user interactions remains peer-to-peer and anonymous.

### Privacy, Usability, and User Adoption

User adoption is a critical factor in the success of any contact tracing app. Our simulation results suggest that if participation is not high, the infection will continue to spread through the population via those not using the app and the intervention will have limited effect. It is worth emphasizing that in certain countries (such as the United States), privacy concerns may present a significant barrier to adoption [17]. Location tracking is a type of data collection that may deter many from using an app, [18] especially when this data is being collected by or shared with government entities [17]. By not requesting access to location data, our app avoids this potential hurdle to adoption.

An additional barrier to entry that may often be overlooked is user registration. By user registration, we simply refer to the process of creating an account to use a web-based service, often by providing an email address and password. Some individuals will refrain from such a process due to concerns with disclosing their email address or other personal information such as their name or home address. For others, the process will become a barrier simply due to the inconvenience associated with the process [19]. Regardless of the motivating factor, user registration is likely to play a role in determining user adoption. Our app’s lack of a user registration process can be expected to improve adoption rates.

### Development Time and Complexity

Two aspects of developing smartphone apps that are often underappreciated are development time and complexity. Complex software not only takes longer to develop, but also is more prone to failures; a noteworthy example was the failed initial launch of the HealthCare.gov website [20]. The COVID-19 pandemic represents a time-sensitive public health crisis, and any technological solution applied to this crisis would need to be developed in a manner that is not only rapid, but also robust. The smartphone app we have proposed, due to its simplistic design, could be deployed swiftly without sacrificing robustness. Additionally, our proposed app would ideally be released under the endorsement or guidance of government entities, especially for the purposes of confirming diagnoses, but it would not require the type of intimate data sharing and coordination that are implied by alternative designs. Thus, government agency overhead would be minimal.

### Manipulation and Fraud

In the first iteration of the app, there was no mechanism for verifying reports of positive diagnoses. Thus, the system was vulnerable to manipulation. We have addressed this issue by building a mechanism for reporting *confirmed* diagnoses. For this, we built a proof-of-concept administrative system that allows authorized users to generate *confirmation codes*. Authorized users could be health care or laboratory workers who process test results or deliver such results to patients. These confirmation codes, which are stored in a database on the server, can be printed by the authorized personnel in the form of QR codes, which are given to patients who receive positive diagnoses. The app allows users to opt for reporting a *confirmed* diagnosis, in which case the user is prompted to scan their printed QR code. The QR code data is sent to the server, where it is checked for validity against its database, and then marked as redeemed, so that it cannot be reused. The associated status positive contact point is marked as confirmed in the server’s transmission graph.

In addition, users of the app are now presented with a setting that allows them to rely solely on confirmed diagnoses, in which case their risk level is calculated based only on *confirmed* status positive contact points. When this setting is enabled, users are protected from fraudulent reports. The app can therefore be used both with and without confirmation of diagnoses, depending on whether the app is used in a setting where generation of confirmation codes has been coordinated with health care and testing facilities.

It is important to note that while this feature requires some additional overhead in the form of coordinating with health care facilities that administer or deliver tests to patients, no patient data is ever used in generating or storing these confirmation codes. The codes are simply random sequences of characters, and authorized users are never prompted or allowed to enter patient data into the system. Thus, user privacy is not compromised with the addition of this feature. Because patient information is not processed through this system, health care or laboratory workers could be authorized liberally through a system of tiered privileges. Should a malicious user gain access to the system, this user would be able to do nothing more than manipulate the app by reporting false confirmed diagnoses.

### Limitations

The smartphone app we propose is not without its limitations and concerns. The primary concern we have identified relates directly to the topic of user adoption. The use of location-based traffic detection algorithms may provide a more robust measure for estimating user location at points of contact, but this practice presents many potential privacy concerns, where some users simply may not be comfortable with an app that tracks their real-time location. Specifically with this app, users will be expected to create contact points by scanning QR codes whenever gathering with other people. This would ideally include not only private gatherings, but also public outings at places such as local businesses. To facilitate contact points for larger numbers of people, local businesses could allow customers to join a contact point by scanning a QR code upon entry. However, users may become fatigued from such behavior over time and choose to discontinue or may be dissuaded from participating at the onset. Under normal circumstances, these hurdles might deter most users; however, due to the tremendous impact of a pandemic, users may be motivated to overlook these inconveniences in light of alternative, more invasive location-tracking measures.

An additional concern is that there is no way to ensure that confirmed diagnoses will be reported through the app. Some users may feel uncomfortable reporting this information through the app, despite the anonymity provided. It should be expected that some fraction of users will not report their confirmed diagnoses, either due to negligence or privacy concerns. However, we have demonstrated through our simulations that a peer-to-peer contact tracing app such as ours can be effective without 100% participation. Thus, as long as a significant fraction of users with a positive diagnosis report through the app, we can expect the system to maintain its efficacy.

Finally, it should be noted that the transmission graph of interactions between individuals may not completely capture all possible routes of transmission, even with complete participation. For example, microbial agents may be transmitted via transportation of objects such as mailed envelopes and packages. Additionally, microbes may linger on surfaces and thus be transferred between individuals who are at the same location at different times. Measures can be taken to mitigate these transmission routes. First, objects and surfaces can be disinfected between interactions to limit transfer beyond the interaction. Second, contact points can be left open for longer periods of time to more accurately capture the possible transmission routes. For example, a grocery store may host a contact point by allowing customers to scan a QR code upon entry. This contact point could be left open from the opening time to the closing time so that all customers shopping that day would be registered into a single contact point, reflecting the fact that an infected shopper in the morning could transfer microbes across surfaces to a shopper that was present in the afternoon. To limit transmission of microbes from one day to the next, stores could thoroughly disinfect surfaces between closing time one day and opening time the next day.

### Comparison With Related Work

The majority of contact tracing and similar surveillance apps rely heavily on the collection of intimate personal data and are not designed to preserve privacy. A popular smartphone app in the United States at the time of this writing is HEALTHLYNKED COVID-19 Tracker. This app differs from ours in two fundamental aspects. First, it is not a contact tracing app. The core feature of HEALTHLYNKED COVID-19 Tracker is a map that broadcasts locations of COVID-19 suspected cases, confirmed cases, and deaths based on a combination of self-reported and WHO-confirmed data. This data alone is insufficient for contact tracing, which requires identification with high specificity of individuals who have interacted with an infected person during some period of time prior to diagnosis. Second, HEALTHLYNKED COVID-19 Tracker requests access to user location, raising privacy concerns. Our peer-to-peer contact tracing app is able to perform detailed contact tracing while protecting user privacy by refraining from requesting personal data such as user location.

To our knowledge, only one prior attempt has been made to develop a privacy-preserving contact tracing app. PrivateKit: Safe Paths was developed in response to the COVID-19 pandemic as a privacy-preserving alternative to more invasive apps. PrivateKit: Safe Paths relies on location data, similar to other contact tracing apps; however, it offers unique protections for user data, such as encryption of location information, which can only be disclosed on a voluntary basis to government officials upon request.

Our app differs notably from PrivateKit: Safe Paths in two respects. First, PrivateKit: Safe Paths attempts to keep user location data private and secure, while our app does not collect this data in the first place, thus minimizing risk and maximizing user confidence. Second, the PrivateKit: Safe Paths app currently relies on intervention from a government entity once a positive diagnosis has been reported, while our app is peer-to-peer in this sense and can function entirely without intervention from any centralized entities, except to validate positive diagnoses. Both PrivateKit: Safe Paths and our peer-to-peer contact tracing app pursue similar objectives, but they differ considerably in their complexity and approach to protecting privacy. We offer that our app provides key advantages in terms of user privacy, simplicity, and minimal dependence on centralized entities, factors that can play a crucial role in responding to a public health emergency such as the COVID-19 pandemic.

An additional recent development is the release of Apple’s official COVID-19 app. This app was developed in coordination with the Centers for Disease Control and Prevention and provides users with resources and guidance relating to the pandemic. In particular, it provides users with a questionnaire regarding symptoms and recent exposures and offers guidance on how to respond, including recommendations for measures such as self-isolation and social distancing, and whether or not testing is recommended. Such an app serves as a valuable resource for iOS users, and future iterations could potentially incorporate features of the app we have proposed here. The Apple COVID-19 app could additionally provide guidance on how to use a peer-to-peer contact tracing app.

### Conclusions

We have proposed a novel peer-to-peer smartphone app for contact tracing that does not use personal data, such as location, and hence preserves user privacy. We have developed a prototype of this app, which is open source and publicly available as well as a computer simulation model that demonstrates the potential of our app to impact the course of a pandemic. Such an app could potentially be applied to the COVID-19 pandemic as well as others in the future to achieve a middle ground between drastic isolation measures and unmitigated disease spread. We hope that our proposal inspires future work in developing technology-based contact tracing solutions that preserve user privacy. Future work may involve testing our app on real devices in either a real or artificial pandemic setting as well as enhancements that build on our approach.

## Abbreviations

COVID-19: novel coronavirus disease 2019
QR: Quick Response
WHO: World Health Organization
